# Reply: No-Reflow During Primary PCI: Pharmacology Over Delivery

**DOI:** 10.1016/j.jacadv.2026.102976

**Published:** 2026-08-26

**Authors:** Federico Oliveri, Lorenzo Tua, Luca Raone, Francesco Maria Sparasci, Jose’ Montero-Cabezas

**Affiliations:** a Leiden University Medical Center, Leiden, the Netherlands; b Cardiology Unit, Fondazione IRCCS Policlinico San Matteo, Pavia, Italy; c University of Pavia, Pavia, Italy

We thank Dr Stracqualursi and colleagues for their insightful comments regarding our network study on intracoronary vasoactive therapies for no-reflow.^1^ While we acknowledge certain limitations, we disagree with the hypothesis that a “drug delivery bias” significantly confounds our results.

First, it has been hypothesized that delivery strategies, including systemic intravenous infusion, could influence the observed results. However, as specified in our methods, studies involving intravenous administration were strictly excluded to ensure that only direct intracoronary pharmacologic effects were evaluated.^1^

Second, for our primary endpoint (restoration of TIMI flow grade 3), between-study heterogeneity was negligible (σ^2^ = 0; I^2^ = 0%). This consistency across 13 randomized controlled trials suggests that the therapeutic benefit is primarily driven by the pharmacologic properties of the agents themselves, rather than by procedural variations in delivery. Importantly, variations in intracoronary delivery techniques are inherently embedded within the randomized evidence included in our analysis. If such differences had a dominant impact on treatment effect, one would expect this to manifest as increased between-study heterogeneity, which was not observed.

Third, in meta-regression analyses exploring potential effect modifiers, including mechanical thrombectomy and the use of glycoprotein IIb/IIIa inhibitors, none of these procedural factors significantly influenced the restoration of TIMI flow grade 3.

Thus, although we agree that future studies should further explore dose optimization and standardized delivery protocols, our findings provide robust evidence to inform drug selection in a real-world clinical scenario previously characterized by heterogeneous data. The high probability that verapamil and epinephrine are the most effective agents remains the central message of our study.
